# Safety of re‐challenging adults with acute lymphoblastic leukemia with PEG‐asparaginase‐induced severe hypertriglyceridemia when treated with a pediatric‐inspired regimen

**DOI:** 10.1002/jha2.607

**Published:** 2022-11-04

**Authors:** Ibrahim Al Nabhani, Claire Andrews, Jad Sibai, Eshetu Atenafu, Taylor Young, Steven M. Chan, Vikas Gupta, Dawn Maze, Aaron D. Schimmer, Andre C. Schuh, Karen Yee, Hassan Sibai

**Affiliations:** ^1^ Division of Medical Oncology and Hematology, Princess Margaret Cancer Centre University Health Network Toronto Ontario Canada

**Keywords:** acute lymphoblastic leukemia, acute pancreatitis, hypertriglyceridemia, PEG‐asparaginase, toxicity

## Abstract

PEG‐asparaginase is used as a treatment for Philadelphia‐negative acute lymphoblastic leukemia. In pediatric studies, triglycerides (TGs) were affected more by PEG‐asparaginase than by native L‐asparaginase (10.0% vs. 5.5%). We conducted a retrospective study to determine the safety of re‐challenging adult patients with PEG‐asparaginase after experiencing an episode of severe hypertriglyceridemia (>1000 mg/dl or 11.4 mmol/L). The incidence of hypertriglyceridemia associated with PEG‐asparaginase in adult patients was high (67.5%). Therefore, checking TGs at baseline and monitoring levels while receiving PEG‐asparaginase need to be considered and studied in prospective studies. However, in patients with hypertriglyceridemia not complicated by acute pancreatitis, re‐challenging is safe once TG levels normalize.

## 1 BACKGROUND

Pegylated‐asparaginase (PEG‐asparaginase), a long‐acting formulation of L‐asparaginase, is used as a treatment for Philadelphia‐negative acute lymphoblastic leukemia (ALL). Its antitumor effect results from the depletion of asparagine, an amino acid essential to leukemic cells, and the subsequent inhibition of protein synthesis, leading to considerable cytotoxicity. In pediatric studies, triglycerides (TG) were affected more by PEG‐asparaginase than by native L‐asparaginase (10.5% vs. 5.5%) [1, 2, 3, 4]. The true incidence of hypertriglyceridemia in adults treated with PEG‐asparaginase as part of a pediatric‐inspired asparaginase regimen is not exactly known. However, several prospective studies of pediatric patients and young adults with ALL have reported a 1%–18% incidence of grades 3–4 hypertriglyceridemia with PEG‐asparaginase [1, 5, 6, 7, 8, 9, 10, 11]. In a study conducted at the Memorial Sloan Kettering Cancer Center, TG levels were measured twice a week for at least 2 weeks following each PEG‐asparaginase dose, regardless of symptoms; grades 3–4 hypertriglyceridemia was observed in 59% of the patients. Multivariate analysis was performed as part of this study and showed that older age and higher PEG‐asparaginase doses were associated with a higher incidence of hypertriglyceridemia [12].

The clinical significance of hypertriglyceridemia and the safety of re‐challenging patients with hypertriglyceridemia with PEG‐asparaginase are unclear. Additionally, there is variability in practice among clinicians in monitoring, treating, and managing the next dose of PEG‐asparaginase in these patients.

## 2 AIMS

We conducted a retrospective study to determine the safety of re‐challenging adult patients treated with PEG‐asparaginase after experiencing an episode of severe hypertriglyceridemia (>1000 mg/dl or 11.4 mmol/L), and we examined how these patients were monitored.

## 3 METHODS

At the Princess Margaret Cancer Center (PMCC), we switched from native *Escherichia coli* asparaginase to PEG‐asparaginase using the modified pediatric Dana Farber Cancer Institute (DFCI) protocols for the treatment of adults with Philadelphia‐negative ALL due to a shortage in the supply of native *E. coli* asparaginase. Currently, we use PEG‐asparaginase for all age groups, with a dose range from 1000 IU/m^2^–1,250 IU/m^2^. Although this dose is similar to many other centers other, however, less than the dose used at other centers (up to 2000 IU/m^2^/dose) to avoid toxicities noted at higher doses specifically hepatoxicities. As the PMCC has the capacity to do levels with each dose of PEG‐asparagine (through and peak) and all patients had targeted activity levels >0.1, and dose can be adjusted to achieve this target, except for <10% who inactivated PEG‐asparaginase silently.

A total of 56 consecutive adult patients who received PEG‐asparaginase as part of the DFCI protocol for first‐line treatment of Philadelphia‐negative ALL between October 2018 and March 2021 at the PMCC were retrospectively reviewed. Forty of these patients had their TG data available during induction and intensification and were analyzed for the incidence of hypertriglyceridemia and their subsequent outcomes after re‐challenging them. In addition, we reviewed the charts to collect data on monitoring strategies and treatments that were given to patients.

During the data collection process, the study used cut‐offs for triglycerides levels as defined by the 2020 consensus statement by the American Association of Clinical Endocrinologists and American College of Endocrinology on the management of dyslipidemia and prevention of cardiovascular disease (normal – <150 mg/dl [<1.7 mmol/L], moderate hypertriglyceridemia – 150–999 mg/dl [1.7 to 11.3 mmol/L], severe hypertriglyceridemia – ≥1000 mg/dl [≥11.4 mmol/L]) [13].

## 4 RESULTS

The median age of the patients was 35 years (19–77 years), and 23 (57.5%) were men. Of the 40 patients, 27 had mild, moderate, or severe hypertriglyceridemia (67.5%), and seven had severe hypertriglyceridemia (17.5%). Moderate (≥5.7 mmol/L) and severe TG levels (≥11.4) occurred a median of 18 days from PEG‐asparaginase treatment (See Table 1).

**TABLE 1:** Characteristics of the patients at baseline (*N* = 40)

Characteristic	
Age, years	Median age, 35 (19–77) Nine patients > 60
Sex	23 males (57.5%)
Body mass index	≤ 24.9 (*N* = 20) 25–29.9 (*N* = 15) ≥ 30 (*N* = 5)
Triglyceride's data collection start date	Induction: 14 Intensification cycle #1: 8 Intensification cycle #2 and beyond: 18 Total of 40/56 have triglycerides data

The number of patients was small; we did not conduct a multivariate analysis. Univariate analysis of the 40 patients with available data based on the age group (>60 vs. ≤60 years), the subtype of leukemia (T cell ALL, mixed phenotype acute leukemia [MPAL], B cell ALL), and body mass index (BMI) (24.9 > vs. ≤24.9) was performed to determine the impact of the above factors on hypertriglyceridemia incidence in this group of patients. There was no difference found based on the age group (>60 vs. ≤60, *p*‐value 0.6904), the subtype of leukemia (T cell ALL, MPAL, and B cell ALL, *p*‐value 0.6181), and BMI (>24.9 vs. ≤24.9, *p*‐value 0.0914) (See Table 2).

**TABLE 2:** Univariate analysis of patients with ALL and hypertriglyceridemia (*N* = 40)

	Incidence of hypertriglyceridemia			
	Mild, moderate or severe (*N* = 27)	Normal (*N* = 13)	Total (*N* = 40)	*p*‐Value
**Age Group**				
>60	7 (25.93%)	2 (15.38%)	9 (22.50%)	0.6904
≤60	20 (74.07%)	11 (84.62%)	31 (77.50%)	
**Subtype of leukemia**				
T cell All	9 (33.33%)	3 (25.00%)	12 (30.77%)	0.6181
MPAL	2 (7.41%)	1 (8.33%)	3 (7.69%)	
B cell ALL	16 (59.26%)	9 (66.67%)	25 (61.54%)	
**BMI**				
>24.9	16 (59.26%)	4 (30.77%)	20 (50.00%)	0.0914
≤24.9	11 (40.74%)	9 (69.23%)	20 (50.00%)	

Abbreviations: ALL, acute lymphoblastic leukemia; BMI, body mass index; MPAL, mixed phenotype acute leukemia.

The management of patients with severe hypertriglyceridemia was supportive in a low‐fat diet, hydration, and fenofibrate or gemfibrozil. The following treatment cycle with PEG‐asparaginase was delayed by 2 weeks on average (range 1–3) until the TG level decreased to at least moderate (≤11.4 mmol/L). None of these patients required plasmapheresis or insulin infusion as a treatment for hypertriglyceridemia. Only one of seven patients with severe hypertriglyceridemia (developed severe hypertriglyceridemia after the first intensification cycle and proceeded to the next cycle before improvement of his TG level) developed acute pancreatitis. The patient was not re‐challenged with PEG‐asparaginase. The remaining six patients were re‐challenged after their TG levels dropped to moderate  without developing pancreatitis. Four of the remaining patients completed their intensification therapy, and two continued to receive PEG‐asparaginase as part of the intensification treatment at the time of this data analysis. Among the remaining 33 patients analyzed, there was only another case of pancreatitis in a patient with mild raise in triglycerides. This patient's etiology of pancreatitis was thought to be due to the direct effect of peg‐asparaginase.

## 5 SUMMARY/CONCLUSION

Peg‐asparaginase is an important drug for the treatment of Philadelphia‐negative ALL. The incidence of severe hypertriglyceridemia was high, and given this high incidence, we adjusted our protocol to check TGs at baseline, administering prophylaxis fenofibrate during induction and intensification. In patients with severe hypertriglyceridemia not complicated by acute pancreatitis, re‐challenging these patients are safe.

Measures, including using, monitoring levels at baseline and during induction and intensification after receiving PEG‐asparaginase to safely receive treatment in subsequent cycles, anti‐TG medications and PEG‐asparaginase dose reductions in subsequent cycles, can help to reduce the incidence of hypertriglyceridemia and its associated complications, although this needs to be further confirmed in larger prospective studies.

## AUTHOR CONTRIBUTIONS

Ibrahim Al Nabhani and Hassan Sibai were involved in conception, design of the study and analysis of the data. Ibrahim Al Nabhani, Hassan Sibai, Claire Andrews, and Jad Sibai were involved in data collection. Ibrahim Al Nabhani and Hassan Sibai drafted the manuscript. All authors participated in data analysis and interpretation of results. All authors critically revised the manuscript and porvided final approval or submission.

## CONFLICT OF INTEREST

The authors declare they have no conflicts of interest.

## FUNDING INFORMATION

The authors received no specific funding for this work.

## ETHICS STATEMENT

This study is in accordance with the declaration of Helsinki for the ethical treatment of human research participants.

## Data Availability

Data can be provided upon request from the corresponding author.
